# Genetic variants in the *HLA‐C* gene may reduce the deleterious effect of APOE4 on Alzheimer's disease: New findings from UK Biobank

**DOI:** 10.1002/alz70855_104042

**Published:** 2025-12-24

**Authors:** Anatoliy I Yashin, Deqing Wu, Hongzhe Duan, Rachel Holmes, Olivia Bagley, Konstantin Arbeev, Svetlana Ukraintseva

**Affiliations:** ^1^ Social Science Research Institute, Duke University, Durham, NC, USA; ^2^ Duke University, Durham, NC, USA

## Abstract

**Background:**

*APOE4* is the strongest genetic risk factor for late‐onset Alzheimer's disease (AD), with a higher effect in females than in males. Identifying factors that may reduce the detrimental impact of *APOE4* on AD is an important research objective. Accumulating evidence points to a key role of the innate and adaptive immune system in AD, including natural killer (NK) and cytotoxic CD8+ T cells infiltrating the Central Nervous System (CNS) in AD, contributing to neuroinflammation and neuronal death. Here, we investigated how polymorphism of the HLA‐C (Human Leucocyte Antigen‐C) gene, a major regulator of NK and CD8+ T cells, may influence AD risk in the presence of the *APOE4* allele in males and females.

**Method:**

We assessed the association of the *HLA‐C* genetic variation in study participants of the UK Biobanks (UKB), who were double carriers of the *APOE4* allele, with AD reported in their mothers and fathers who carried at least one *APOE4* allele (1562 and 798 in the case and 7572 and 8336 in the control groups, respectively). The analysis used a logistic regression model in the REGENIE and was repeated using the PLINK2 software package. The PLINK2 clumping procedure was used to reduce the number of statistical tests.

**Result:**

The SNPs rs2074488 and rs2074491 in the HLA‐C region, genotyped in UKB participants, were significantly associated with lower chances of AD in mothers who carried at least one copy of the APOE4. Three SNPs in the HLA‐C region (+/‐2KB): rs13203722, rs13218306, and rs12207404 showed deleterious associations with AD in mothers. Both analyses (using PLINK2 and REGENIE) supported these results. The protective SNPs and the deleterious ones were not in linkage disequilibrium. A similar analysis did not produce significant associations of HLA‐C genetic variants with AD in fathers.

**Conclusion:**

The detected significant associations of *HLA*‐*C* genetic variants in offspring with AD odds in mothers carrying at least one *APOE4* allele suggest that *HLA*‐*C* may modulate (attenuate or aggravate) the impact of APOE4, the strongest genetic risk factor, on AD occurrence. This finding further supports the role of infections and other factors initiating immune response in AD.

